# Accounting for immunoprecipitation efficiencies in the statistical analysis of ChIP-seq data

**DOI:** 10.1186/1471-2105-14-169

**Published:** 2013-05-30

**Authors:** Yanchun Bao, Veronica Vinciotti, Ernst Wit, Peter AC ’t Hoen

**Affiliations:** 1 School of Information Systems, Computing and Mathematics, Brunel University, London, UK; 2 Institute of Mathematics and Computing Science, University of Groningen, Groningen, The Netherlands; 3 Department of Human Genetics, Leiden University Medical Center, Leiden, The Netherlands; 4 Netherlands Bioinformatics Centre, Nijmegen, The Netherlands

## Abstract

**Background:**

ImmunoPrecipitation (IP) efficiencies may vary largely between different antibodies and between repeated experiments with the same antibody. These differences have a large impact on the quality of ChIP-seq data: a more efficient experiment will necessarily lead to a higher signal to background ratio, and therefore to an apparent larger number of enriched regions, compared to a less efficient experiment. In this paper, we show how IP efficiencies can be explicitly accounted for in the joint statistical modelling of ChIP-seq data.

**Results:**

We fit a latent mixture model to eight experiments on two proteins, from two laboratories where different antibodies are used for the two proteins. We use the model parameters to estimate the efficiencies of individual experiments, and find that these are clearly different for the different laboratories, and amongst technical replicates from the same lab. When we account for ChIP efficiency, we find more regions bound in the more efficient experiments than in the less efficient ones, at the same false discovery rate. A priori knowledge of the same number of binding sites across experiments can also be included in the model for a more robust detection of differentially bound regions among two different proteins.

**Conclusions:**

We propose a statistical model for the detection of enriched and differentially bound regions from multiple ChIP-seq data sets. The framework that we present accounts explicitly for IP efficiencies in ChIP-seq data, and allows to model jointly, rather than individually, replicates and experiments from different proteins, leading to more robust biological conclusions.

## Background

ChIP-sequencing, also known as ChIP-seq, is a recently established technique to detect protein-DNA interactions in vivo on a genome-wide scale [1]. ChIP-seq combines Chromatin ImmunoPrecipitation (ChIP) with massively parallel DNA sequencing to identify all DNA binding sites of a Transcription Factor (TF) or genomic regions with certain histone modification marks. The ChIP process captures cross linked and sheared DNA-protein complexes using an antibody against a protein of interest. After decrosslinking of the protein-DNA complexes, the final DNA pool is enriched in DNA fragments bound by the protein of interest, but there are always random genomic DNA fragments piggybacking on the specific DNA fragments. The degree of enrichment depends on the ChIP efficiency. A more efficient experiment will induce a higher proportion of protein-bound fragments in the mixture pool, and generate more sequence reads in bound regions and less sequence reads in non-bound regions, than an experiment with lower ChIP efficiency. As a result, the more efficient experiment will have more power to discriminate between bound and non-bound genomic regions and generally show a larger number of bound regions.

The antibody used is the most critical factor affecting ChIP efficiency [2]. However, different ChIP efficiencies are also observed between different batches when using the same antibody, since ChIP protocols are notoriously difficult to standardize and control. In general, we may encounter three relevant scenarios where differences in ChIP efficiencies play a role: (i) the comparison of bound regions between two experimental conditions subjected to ChIPs with the same antibody but with variable efficiencies; (ii) the comparison of bound regions of the same TF or marked with the same histone modification but profiled with different antibodies; (iii) the comparison of bound regions from two different TFs or marked with different histone modifications, profiled with different antibodies. When making comparisons without considering the ChIP efficiencies, the number of overlapping regions may be underestimated while the number of differentially bound regions may be overestimated. A number of methods have been proposed recently for comparative analyses of ChIP-seq data e.g. [3-9]. In general, there is recognition in the literature of different specificities associated to different antibodies used in ChIP-seq experiments, e.g. [2], and attempts are made to account for these in the analysis. These are often in the form of a pre-selection of regions for the analysis: in [3,6] only regions with high signal to background ratios are used for further analyses and normalization procedures, in [7] the normalization is performed only on commonly enriched regions. A control experiment is often used to aid the detection of truly enriched regions (e.g. in PeakSeq [10] and W-ChIPeaks [11]). However, overall, there is a shortage of formal definition of ChIP efficiency and a limited focus on how this affects the interpretation of the results and how this should be fully accounted for in the statistical analysis of the data and consequently in the detection of enriched and differentially bound regions. In this paper, we address these issues using ChIP-seq data from a number of experiments conducted by different laboratories on two highly similar but different proteins.

P300 and the CREB binding protein (CBP) are two Histone AcetylTransferases (HATs) which are transcription co-activators for a broad range of genes involved in various multiple cellular processes. P300 and CBP have highly similar roles in transcriptional activation, but also differ in some aspects that are still not fully understood [12]. This is reflected by the large but incomplete overlap in p300 and CBP binding sites in the genome [13,14]. In the ChIP-seq study of [14] it is known that the antibody specificity for the p300 experiments is higher than for the CBP experiments. Using a Fisher exact test, [14] find that the number of regions preferentially bound by p300 is largely greater than the number of regions preferentially bound by CBP. In [13], two experiments are conducted on the same two proteins, but using a different cell line. In this case, the antibody specificity for the CBP experiment is known to be higher than the one for p300. Consequently, the number of regions preferentially bound by p300 found by this study is much smaller than the number of regions associated only with CBP. Despite the different experimental set-ups of the two studies, these results suggest that the differences in ChIP efficiencies associated with the antibodies used can have a major impact on the findings of regions that are differentially bound by CBP or p300, and may mask the real heterogeneity between the two HATs and the two cell types studied. Hence, there is a need to explicitly account for these in the statistical analysis and interpretation of the results.

A large number of statistical methods have been developed in the last few years for modelling ChIP-seq data. The majority of these concentrate on the detection of peak-type profiles such as the ones generated by DNA-binding TFs. Some others are proposed for detecting genomic regions with broader signals such as those bound by RNA Polymerase II binding [4] or marked with specific histone modifications [15,16]. If no control experiment is available (e.g. a ChIP experiment with a non-specific IgG control antibody), a general strategy is to model the background read distribution and then assign a statistical significance cut-off for the detection of candidate peaks or enriched regions using either analytical or simulation approaches. One popular model for the background is given by the Poisson or Negative Binomial (NB) distributions, which are used by a number of available software packages (FindPeak [17], USeq [18], CisGenome [19], SISSRs [20]). An alternative to the global Poisson or NB models is to use local Poisson models (e.g. MACS [21] and ChIPseqR [22]), mixture of Poisson/NB models (e.g. MOSAiCS [23]) or more advanced hidden Markov mixture or random field models (e.g. BayesPeak [24], HPeak [25] and iSeq [26]).

In this paper, we use a latent mixture model, as described in the Methods section, and show how this model accounts for the ChIP efficiency of an experiment, by modelling an appropriate signal to background ratio. The general idea is that the different components of the mixture model give flexibility to model both well separated signal and background components (i.e. efficient experiments) and more overlapping components (i.e. less efficient experiments). A formal definition of ChIP efficiency is given, which can be easily extended to mixture models of more than two components. Therefore, other methods based on mixture modelling, such as the ones mentioned above, could be used within the same framework described in this paper. The fact that different experiments, even technical replicates from the same lab, can have different IP efficiencies has probably been the main reason why, to date, statistical modelling of ChIP-seq data sets, and corresponding implementations, have been developed for individual experiments. In the presence of technical or biological replicates, the results from the different analyses are subsequently combined to increase the robustness in the detection of regions and circumvent the problem of different signal to background ratios [7]. One major contribution of this paper is to show how a mixture model framework that explicitly account for ChIP efficiencies can be used to perform a joint analysis of ChIP data from multiple experiments on different proteins, aiding to a more robust detection of enriched and differentially bound regions.

## Results and discussion

### Joint modelling of ChIP-seq data with multiple replicates and different IP efficiencies

The analyzed material from the immunoprecipitation step of a ChIP-seq experiment is always a mix of fragments bound by the transcription factor (true signal) and random background fragments (background signal). Furthermore, the majority of regions in the genome is not enriched and should therefore contain only background signal. We would generally expect that the bin counts reflect this mixture pattern. That is, some bins are enriched regions with a lot of tags (possibly a ’peak’ for TF binding) and most other bins are not enriched, containing only few tags. This motivated us to assume a mixture model framework for the counts. The model that we present in this paper does not make any use of peak information and is therefore more suitable for the detection of broad regions, such as those marked with histone modifications.

Let *M* be the total number of mappable bins and *Y*_*mcji*_ the counts in the *m*th bin, *m* = 1,2,⋯,*M*, under condition *c*, antibody *j* and replicate *i*. In our context, the condition *c* stands for a particular protein (either CBP or p300) at a particular time point, and *i* = 1,…,*n*_*j*_ is the number of technical or biological replicates for antibody *j* used in this condition, with *j* = 1,…,*J*. The counts *Y*_*mcji*_ are either from a background population (non-enriched region) or a from a signal population (enriched region). Let *X*_*mc*_ be the unobserved random variable specifying if the *m*th bin is enriched (*X*_*mc*_ = 1) or non-enriched (*X*_*mc*_ = 0) under condition *c*. Clearly, this latent state does not depend on ChIP efficiencies. Similarly to the model used in MOSAiCS for single experiments [23], we define a joint mixture model for *Y*_*mcji*_ as follows:

$$\begin{array}{ll} Y_{\text{mcji}}\;∼\;p_{c}f(y-k_{\text{cji}}|\theta_{\text{cji}}^{S})+(1-p_{c})f(y|\theta_{\text{cji}}^{B}),\; & \; \end{array}$$

where *p*_*c*_ = *P*(*X*_*mc*_ = 1) is the mixture portion of the signal component and $f(y,\theta_{\text{cji}}^{S})$ and $f(y,\theta_{\text{cji}}^{B})$ are the signal and background densities for condition *c*, antibody *j* and replicate *i*, respectively.

Using a mixture model allows to split the signal and background component in the data: this is particularly important when different ChIP efficiencies are observed, as these will induce a different signal to background ratio. The different parameters of the mixture components will allow to capture the different IP efficiencies of individual experiments, whereas the parameter *p*_*c*_, which does not depend on the ChIP efficiencies, allows to properly combine technical and biological replicates with the same or different antibodies. This is not normally done in the literature, rather different analyses are performed for different experiments and the detected regions are further combined at a second stage, e.g. [5,6]. The constant *k*_*cji*_ is a non-negative value that represents the minimum observable tag count in an enriched region and is used to provide greater flexibility to the two-component mixture model, particularly in the presence of a large proportion of zeros. [19,23] set this offset equal to some pre-specified value and use the same value for all experiments. However this assumption does not seem to be supported by the data, where the value of the offset *k* may also depend on the library size and on the different signal and background ratios of the experiments. We therefore opted to keeping this parameter free in our maximum likelihood procedure and estimating it from the data.

We fit this model to the p300 and CBP datasets described in the Methods section, using the EM-procedure outlined in the same section for parameter estimation. The input to the model is count data from all ChIP-seq datasets considered, together with information on which experiments are replicates. The output of the model is the estimates of all the parameters, that is *p*_*c*_, $\theta_{\text{cji}}^{S}$ and $\theta_{\text{cji}}^{B}$ for all *c*, *j* and *i*. The eight experiments considered in this paper are performed by two different labs. In [14], two technical replicates are conducted at time 30 for each of the two proteins. In [13], single experiments are conducted for non-activated T-cells. Given the different cell lines used in the two studies, the experiments from the two different labs cannot be considered as biological replicates. However, the framework described in this paper would be flexible enough to allow for the situation when different replicates are conducted in different labs (and using different antibodies).

Table 1 gives the parameter estimates of the mixture of two NB distributions, using the joint modelling approach just described. The use of NB distributions returned a better fit than a Poisson mixture model in terms of the Bayesian Information Criterion (BIC) values (data not shown here). The second column reports the value of the parameter *p*_*c*_, that is the probability of enrichment. This is the same for technical replicates, as constrained by the model since these are assumed to share the same binding profile. Columns 3 to 6 report the parameters of the mixture distributions. These vary significantly between different experiments, to reflect the different IP efficiencies. Column 7 shows different estimates of the parameter *k* for the eight experiments, suggesting that setting this value fixed a priori, as in [19,23], is generally not advisable.

**Table 1:** **Fitting results by mixture of two negative binomial distributions: mixture parameter estimates (second to fifth column), offset value *k* (sixth column), corresponding estimate of ChIP efficiency (IPE; seventh column) and number of enriched regions at a controlled 0.1% FDR (last column)**

**Experiment**	***p***_***c***_	***μ***_***S***_	***ϕ***_***S***_	***μ***_***B***_	***ϕ***_***B***_	***k***	**IPE**	**# Enriched regions**
CBPT0	0.0305	3.7318	0.6635	1.2788	1.8891	2	0.8973	2383
CBPT301	0.0568	4.5659	1.1781	1.4140	2.7159	2	0.9221	41606
CBPT302	0.0568	8.4491	0.5236	1.1634	1.1867	3	0.9630	
p300T0	0.0414	7.3513	0.7772	1.4159	2.0733	3	0.9628	22250
p300T301	0.0511	7.3276	0.7390	1.3524	3.0402	3	0.9684	65768
p300T302	0.0511	13.9161	0.5700	0.9740	0.9770	3	0.9793	
Wang CBP	0.0180	24.7877	0.3742	4.8347	3.3128	9	0.9621	10251
Wang p300	0.0143	6.0192	0.2438	2.2001	4.3590	4	0.9156	3881

### Quantifying IP efficiencies of ChIP-seq experiments

The mixture model that best fits the data can be further used to derive an estimate of IP efficiency of a ChIP-seq experiment. In the literature, this is often done using informal ad-hoc measurements, e.g. [27] estimate ChIP efficiency by the ratio of hybridization values at the top 1% of bound sites to the bottom 10%, which are taken to represent background levels of binding, whereas [28] measure it using the relative level of protein binding with respect to control regions. In general, ChIP efficiency is often thought in terms of a ratio between the total number of counts in the enriched regions versus the total number of counts in the background regions. In the context of our paper, such a quantity can be estimated by taking the ratio of the expected counts in the signal regions, *μ*_*S*_, versus the expected counts in the background regions, *μ*_*B*_. However, such a measure would not account for overdispersion, or, in general, for more complex distributions of the background and signal components. For this reason, we present a more general measure of IP efficiency in terms of separation of the signal and background components of the mixture model. An efficient experiment will generate well separated signal and background components, whereas a less efficient experiment will generate two more overlapping components. In the Methods section, we provide a formal derivation of this IP efficiency estimate.

Table 1 reports the corresponding IP efficiencies for the eight experiments on p300 and CBP. These estimates reflect existing knowledge on the specificities of the antibodies used for the different proteins, e.g. the efficiencies of the experiments for p300 by [14] are larger than the ones for the CBP experiments, whereas the opposite is observed for the experiments by [13]. Furthermore, it is interesting to note quite a large difference in ChIP efficiency for technical replicates in the study of [14], which is reflected also in the parameter estimates (e.g. differences in the signal and background means for the CBP technical replicates). These different ChIP efficiencies, if not accounted for, can potentially lead to erroneous biological conclusions.

### Accounting for ChIP efficiencies in the detection of enriched regions

ChIP efficiencies need to be properly accounted for in the detection of the regions bound by a protein from the available ChIP-seq data. After fitting a mixture model to count data, the estimates for all the parameters in the model, that is *p*_*c*_, $\theta_{\text{cji}}^{S}$ and $\theta_{\text{cji}}^{B}$, are used to select the regions enriched by p300 and CBP, respectively. A common procedure for mixture models is to set a cut-off on the posterior probabilities of non-enrichment, $P(X_{\text{mc}}=0|y,{\hat{\Theta }}_{\text{cji}},{\hat{k}}_{\text{cji}},{\hat{p}}_{c})$ for regions *m* and condition *c*. We choose this threshold using a controlled False Discovery Rate (FDR) of 0.1*%*, as detailed in the Methods section. The last column of Table 1 gives the number of enriched regions for each condition, in terms of the 1000 bp windows used in the analysis. As technical replicates are modelled jointly, a single list of enriched regions is detected for these experiments.

The important step in the detection of enriched regions is that, in order to properly account for the different ChIP efficiencies, the enriched regions are selected after controlling for the same FDR amongst the different experiments. As shown in Table 1 more regions are detected for the more efficient experiments, as one would expect. For example, the ChIP efficiency of Wang CBP is larger than Wang p300 and this results in more than twice the number of enriched regions detected in the CBP experiment than in the p300 experiment. This should not be confused with the actual number of true binding sites, which is unknown and is better reflected in the estimates of *p*_*c*_. For example, WangCBP is a more efficient experiment than CBPT0, so more regions are detected as enriched in WangCBP than CBPT0 at the same FDR, but the estimated probability of a region being enriched is larger in CBPT0 (*p*_*c*_ = 0.0305) than in WangCBP (*p*_*c*_=0.0180). To emphasize the importance of using the same FDR in the presence of different ChIP efficiencies, Table 2 gives the estimated FDR when we select the same number of enriched regions in the eight experiments. In particular, we consider the case where for each experiment we select the top 31689 regions, which is the average of the number of enriched regions amongst the eight experiments on CBP and p300, and the case where we select 65768 regions, where the most efficient experiment shows acceptably low FDRs. As expected, the more efficient experiments show lower FDRs. This means that not accounting for ChIP efficiency, which we mimic here by assuming a fixed number of enriched regions in all experiments, will result in a greater number of false negatives for the more efficient experiment and a greater number of false positives for the less efficient experiment.

**Table 2:** FDR values when the same number of enriched regions is assumed for all eight experiments

**Experiment**	**31689 bound regions**	**65768 bound regions**
CBPT0	34.21%	56.35%
CBPT30	0.01%	1.70%
p300T0	1.18%	16.22%
p300T30	2.08e-06%	0.10%
WangCBP	26.81%	57.58%
Wangp300	59.94%	77.24%

**Figure 1:**
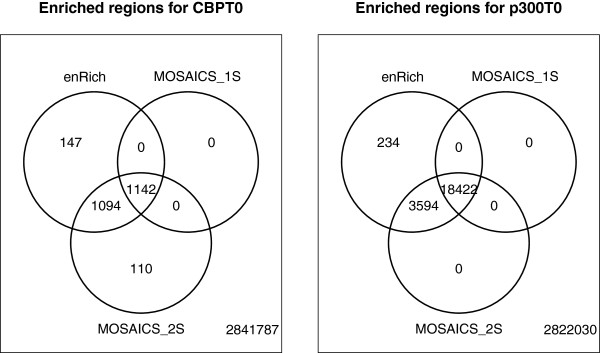
**Venn diagrams of enriched bins detected by enRich and MOSAiCS.** Venn diagrams of enriched bins detected by our method (enRich) and a similar existing method (MOSAiCS) for two selected experiments, CBP at time 0 and p300 at time 0, at a controlled 0.1% FDR. Two versions of MOSAiCS are considered: MOSAiCS_1S fits a mixture model with one background and one signal component, MOSAiCS_2S uses a mixture of two densities for the signal component.

One strength of the approach proposed in this paper is in the fact that replicates are joined in the model by a common assumption of shared binding profiles. This is an assumption on the latent states, prior to the collection of data. The different IP efficiencies of the replicates are further captured by the individual parameters of the signal and background distributions. This joint modelling approach makes an appropriate use of replicates and is expected to return a more robust set of the regions bound by a protein. In the first instance, we compare our results with those from an existing method on single experiments. In particular, we perform a comparison with MOSAiCS [23], which is in spirit very similar to our mixture modelling approach. Figure 1 shows Venn diagrams of the detected regions at the same FDR, for two representative experiments. We compare our approach, denoted as enRich, with two versions of MOSAiCS: MOSAiCS_1S corresponds to a mixture model with one background and one signal component, whereas MOSAiCS_2S fits a mixture of two densities for the signal component. Figure 1 shows how MOSAiCS_2S identifies more bins than MOSAiCS_1S, as expected from a more flexible approach, and how enRich has a very high overlap with MOSAiCS_2S. Despite our method using only one signal component, the maximum likelihood procedure that we use for parameter estimation returns better estimates than the moment estimators used by MOSAiCS. The MOSAiCS_1S model fits an extremely large variance for the signal component to capture the long tail of the distribution of counts. This problem is attenuated by the use of the second signal component.

Having established a very high overlap between our method and an existing approach for single experiments, we now assess the advantages of the joint modelling approach when replicates are available. Table 3 compares the number of regions detected by our approach with the number of regions that are detected at the same FDR by fitting separate mixture models for each of the two replicates and then finding the regions that are detected as enriched by both experiments, which is the common procedure adopted in the current literature, e.g. [6,7,22,29].

**Table 3:** Binding sites for Ramos T30 using separate models for replicates and taking the intersection (top) and the union (bottom) of regions identified by individual analyses at an 0.1% FDR (column 2), compared to a joint analysis of replicates at the same FDR (column 3)

**Experiment**	**Identified using the intersection**			**Additionally identified**		
	**of separate models**			**using joint model**		
	Number	Number	%	Number	Number	%
		containing	containing		containing	containing
		TSS	TSS		TSS	TSS
CBPT301 & CBPT302	5903	1444	24.46%	9659	1942	20.11%
p300T301 & p300T302	22984	5926	25.78%	9861	2676	27.14%
	**Identified using the union**			**Additionally identified**		
	**of separate models**			**using joint model**		
	Number	Number	%	Number	Number	%
		containing	containing		containing	containing
		TSS	TSS		TSS	TSS
CBPT301 & CBPT302	22762	4601	20.21%	18844	3870	20.54%
p300T301 & p300T302	43003	10156	23.62%	22765	1786	7.85%

When conducting separate analyses, different latent profiles *X*_*mc*_, and consequently different *p*_*c*_, are implicitly assumed for each replicate. This goes against reasonable assumptions, as replicates are made under the same condition *c*, and it has the result of reducing the power in the detection of commonly enriched regions. In our comparison, we find that all regions detected by the separate approaches are detected also by the joint modelling approach. On the other hand, it is clear from Table 3 how many more regions are detected when technical replicates are modeled jointly, as in the approach proposed in this paper, rather than individually. Furthermore, when taking the intersection of lists of regions detected by single experiments using a controlled FDR, it is not clear what the level of FDR of the resulting list of regions is. In general, this is expected to be much smaller than the FDR cutoff chosen for each individual experiment, although this is rarely discussed in the literature [30] and shows a further disadvantage of performing individual analyses of replicates. In an attempt to perform a fair comparison with our joint modelling approach, we estimate the FDR of the commonly enriched regions detected by separate experiments using

$$P(X=1|Y_{1},Y_{2})=\frac{P(Y_{1},Y_{2}|X=1)P(X=1)}{P(Y_{1},Y_{2})}$$

for two replicates *Y*_1_ and *Y*_2_, sharing the latent binding profiles *X*, where we estimate the posterior probability *P*(*Y*_1_,*Y*_2_|*X*) from the two separate analyses and we take *P*(*X* = 1) as an average of the two estimates from the two separate analyses. When setting an 0.1*%* FDR cutoff on each individual analysis, this method returns an estimated FDR of 4.0*e* −8 and 4.5*e* −8 for CBP and p300, respectively, for the commonly detected regions. We use these FDR values for the joint modelling results of Table 3 (top). Note that these values are smaller than the 0.1% cutoff chosen for Table 1, thus returning a smaller number of enriched regions for the joint modelling approach. Similar results are obtained by taking the union of separate analyses, rather than the intersection, that is by considering regions that are detected by at least one of the two separate analyses (Table 3, bottom). The FDR of the union of regions is similar to that of individual experiments, but the joint modelling approach consistently finds many more regions than the separate analyses.

**Figure 2:**
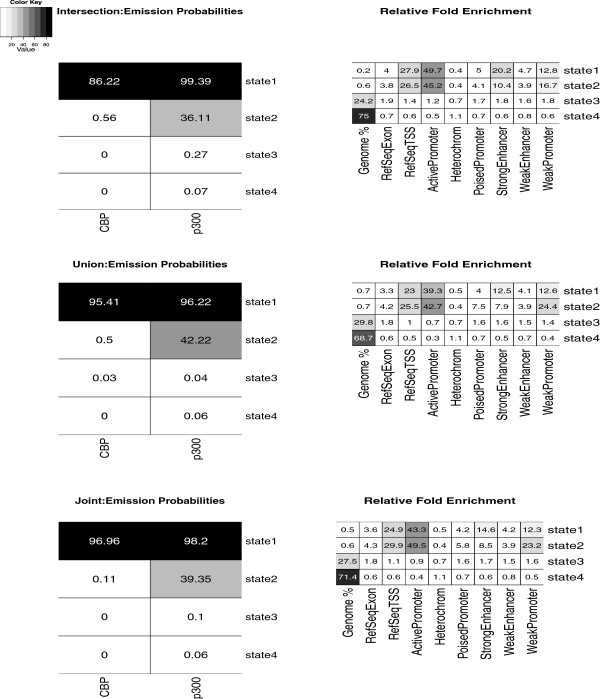
**Validation of enriched bins detected by separate and joint models, using ChromHMM. **Validation of the enriched bins detected by the intersection (CBP: 5903, p300: 22984, first row) and union (CBP: 22762, p300: 43003, second row) of separate analyses of technical replicates at T30, and by the joint model proposed in this paper (CBP: 15562, p300: 32845, last row), using ChromHMM with a 4-state hidden Markov model. The left plots show heatmaps of the probabilities (in percentages) that the CBP and p300 detected bins are enriched given each identified chromatin-state. The right plots show the relative percentage of the genome represented by each chromatin state (first column) and the relative fold enrichment for several types of annotation (remaining columns).

CBP and p300 both have roles in transcriptional activation. To analyze whether the additionally identified CBP and p300 bound regions are not merely false positives but likely functional in transcription activation, the regions are also evaluated for the presence of TSSs of annotated genes (Table 3). With the exception of the last comparison, where an unusually low percentage is observed, these results show that the additionally identified regions have a similar percentage of TSSs to the ones in the independent modelling approach, providing some evidence that these regions are not just noise but genuine binding sites. We use ChromHMM [31] to validate this further and to explore whether other chromatin features are enriched in the regions identified by the different methods. Figure 2 shows the results of ChromHMM using a 4-state hidden Markov model on the enrichment profile given by the intersection and union of separate analyses, each at an 0.1% FDR (first and second row, respectively), and by the joint model (third row), at the same FDR as the intersection. The data from both proteins is jointly modelled by ChromHMM. The left plots give the emission probabilities for the different analyses, that is the probability of the observed enrichment given each of the four possible states. These plots show how, for all analyses, two of the four states explain most of the enrichment pattern in the identified lists. The right plots give the relative fold enrichment for several annotations. These plots show how these two states are mostly enriched with TSSs, active and weak promoters, and weak enhancers. Furthermore, the plots show how the second state, which is enriched only in p300, reflects mainly the different degrees of enrichment of CBP and p300 for the same chromatin features. This is most likely the result of the different ChIP efficiencies of the p300 and CBP experiments, respectively, which result in a larger number of enriched regions for p300 than for CBP and which are not accounted for in ChromHMM. The findings from ChromHMM seem to be consistent across the different analyses. Together with the results in Table 3, one can conclude that by combining replicates jointly at the modelling stage, rather than at a later stage, many more regions are found at the same FDR, and that these regions are generally of the same quality as those found by the individual modelling approach.

### Detection of differentially bound regions

When we have data on two or more proteins, or on one protein and a control, an interesting question is to find the regions that are differentially bound by the two proteins of interest. These are the regions with a large difference in the probabilities of enrichment, *P*(*X*_*mc*_=1|*y*) for the two proteins. Antibody efficiencies also play a role in this as, generally, one would expect to find many regions preferentially bound by a protein for which a more efficient experiment is conducted, than for a protein from the less efficient experiment, simply down to the two different antibodies used. Indeed, this is the case for the two studies by [13,14] mentioned in the introduction. In the literature, techniques which can detect peaks or enriched regions for a single experiment against a control, e.g. MACS [21], ChIPDiff [32] or MOSAiCS [23], can also be used to detect differentially bound regions for two proteins. Here, the general procedure is to use the experiment from the other protein as a control. However this method lacks formal probability definitions on the difference between the two experiments. Furthermore, it is not implementable for those peak-finder methods that do not use control information. More recent methods, such as ChIPnorm [6], allow to compare two experiments on two proteins at the same time, but somewhat sidestep the issue of different IP efficiencies by focussing on regions with high signal to background ratio and normalizing the counts on these regions only. Finally, one of the latest methods, DBChIP [5], allows the inclusion of biological replicates in the model, but does not account for their different IP efficiencies in the detection of enriched and differentially bound regions.

In this paper, we formally develop a test for the detection of differentially binding regions from a number of ChIP-seq experiments on two proteins, based on the statistical model proposed in this paper. The novelty of this test is in the fact that the information from multiple experiments is shared at the modelling stage, by properly accounting for the different IP efficiencies, and is then fed into the test. We consider the following probability of differential binding

(1) $$\begin{array}{ll} \;P(X_{m1}\neq X_{m2}) & =P(X_{m1}=0|Y_{1})P(X_{m2}=1|Y_{2})\;\\ & +P(X_{m1}=1|Y_{1})P(X_{m2}=0|Y_{2})\; \end{array}$$

where *P*(*X*_*mc*_ = 0|*Y*_*c*_) is the probability that the *m*th bin is enriched for protein *c*, estimated by the model described above and from all the data on protein *c*. In this way, all replicates under the same condition are considered in the estimation of the posterior probabilities, returning a more robust set of differentially bound regions.

**Figure 3:**
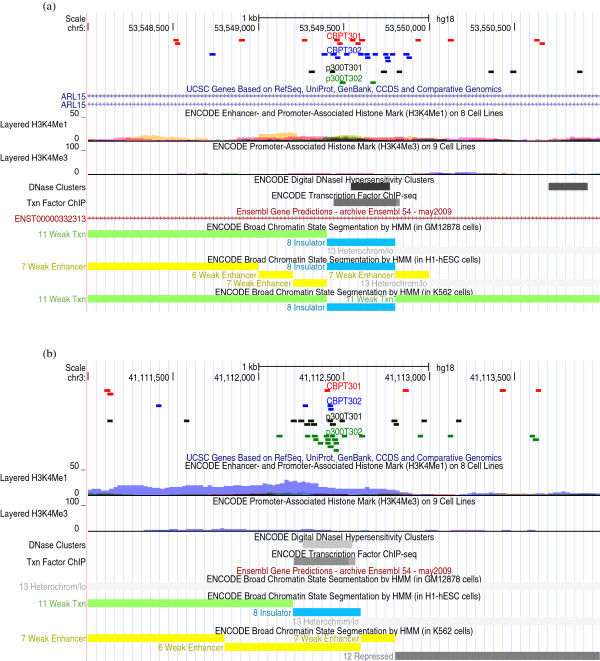
**Genome browser views of representative differentially bound regions.** Genome browser views of two representative regions, which are found differentially bound by CBP **(a)** and p300 **(b)**, using the joint modelling approach but not using the individual modelling approach. The blocks in each figure represent individual sequence reads in CBPT301 (red), CBPT302 (blue), p300T301 (black) and p300T302 (green). Additional evidence is provided from the ENCODE data on H3K4Me1 methylation marks, DNAseI hypersensitive sites and transcription factor binding, and from the Broad annotation of enhancers, promoters and actively transcribed genomic regions in GM12878, hESC and K562 cells. Region **(a)** is in the first intron of ARL15. We observe an overlap with CTCF binding and DNaseI hypersensitivity sites. Region **(b)** is 100 kb upstream of CTNNB1. It is likely functioning as a promoter or enhancer, as suggested by the annotation of histone marks provided by the ENCODE consortium and the Broad Institute.

Table 4 reports the results of this analysis, for the detection of the regions that are bound only by CBP or p300 at 5% FDR, using the parameters estimated by the joint mixture model (Table 1) to compute the posterior probabilities in Equation (1). It is clear how more regions are detected as bound by the protein where more efficient experiments are conducted than for the other protein (i.e. p300 for the Ramos study and CBP for Wang). This is to be expected as there is more power in the detection of these regions, but it should not be misleading: the controlled FDR guarantees that only a controlled number of errors is computed in the detection of regions for either of the two proteins. Only by increasing the FDR even further, would one be able to recover more regions in the less efficient experiments, albeit with a higher probability of false detections. Finally, it is interesting to note how the number of differentially bound regions is more balanced for the case when technical replicates are available (T30). This suggests that properly accounting for replicates at the modelling stage is expected to give more power also in the detection of differentially bound regions. In support of this, we have performed separate mixture modelling analyses for p300T301 versus CBPT301 and p300T302 versus CBPT302 and have taken the union of the differentially bound regions from these two separate analyses. These results are reported in Table 4 and show a remarkable difference with the results from the joint analysis, especially for the less efficient experiment, where many more regions are detected as differentially bound using the joint modelling approach. Figures 3a and 3b give genome browser views of two representative regions that are found differentially bound by CBP and p300, respectively, using the joint modelling approach, but that are not found using the individual modelling approach. These plots show how the power in the detection of differentially bound regions increases when the counts of individual experiments on technical replicates are modelled jointly. Future work will look at validating these regions biologically.

**Table 4:** Number of differentially bound regions at 5% FDR; at T30, where technical replicates are available, the results are given both for the case where the joint model of replicates is used (first column) and for the case where the union of two separate analysis is used (CBPT301 versus p300T301 and CBPT302 versus p300T302, second column)

**Conditions**	**# Regions bound only by CBP**		**# Regions bound only by p300**	
Wang	3069		142	
T0	9		2726	
	Joint analysis	Separate	Joint analysis	Separate
T30	6126	118	9843	3402

**Figure 4:**
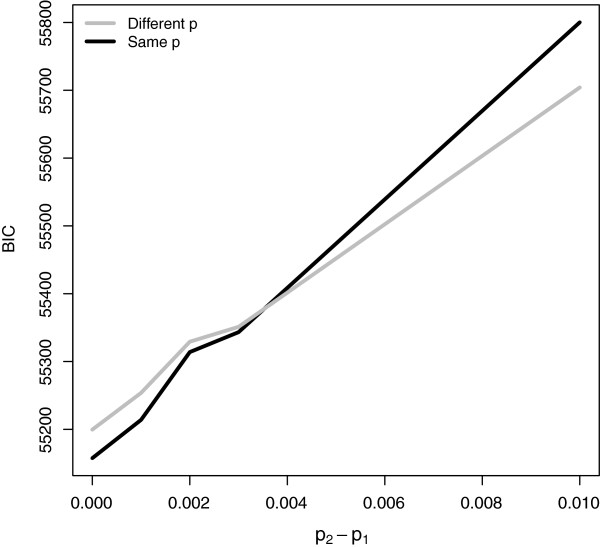
**Simulation study to assess the usefulness of BIC in deciding whether two proteins have equal probability*p***_***c***_**.** BIC values for the model that assumes different *p*_*c*_ probabilities for each condition (grey) and the one that assumes the same probabilities (*p*_1_ = *p*_2_, black). The x-axis shows the true *p*_2_−*p*_1_ values for simulated ChIP-seq data on two experiments with different IP efficiencies.

In the presence of two different proteins, a priori biological knowledge about the two proteins can be further included in the test. In particular, in the context of the model described in this paper, one can impose the assumption that the two proteins have the same number of binding sites, that is *p*_1_=*p*_2_, where *p*_*c*_ is the probability of a region being enriched by protein *c*. If realistic, this assumption is expected to lead to a more robust detection of the enriched regions, by providing a better estimate of the expected number of enriched regions in the different experiments. Indirectly, this allows to better account for the different IP efficiencies of the different experiments. The constraint of *p*_1_ = *p*_2_ can be imposed in the maximum likelihood procedure, in a similar way to parameter estimation in the presence of replicates. However, in this case, we do not make an assumption of equal binding profiles (i.e. *X*_*m*1_ = *X*_*m*2_ for all regions *m*), which is instead appropriate for technical replicates. The main difficulty in implementing this method is in assessing whether the assumption of a same *p*_*c*_ is appropriate in a particular biological context. When no definite knowledge is available, we suggest to compare the fit of a model which makes an assumption of *p*_1_ = *p*_2_ with a model which does not make this assumption. As the two models have a different number of parameters, we suggest to compare them in terms of their BIC value. This is defined in the usual way by $-2\ln L(\hat{\Theta })+r\ln(M)$, with $\hat{\Theta }$ the estimated parameters in the model, $L(\hat{\Theta })$ the maximum likelihood and *r* the number of parameters. The estimated parameters are different depending on whether the constraint of equal *p*_*c*_ is imposed or not, and the best model is chosen as the one with the lowest BIC. Figure 4 shows the output of a simulation study where we have assessed whether this BIC measure leads to an informative choice in our context. We have simulated count data on 10000 regions for two different experiments (e.g. proteins), using the mixture distributions *p*_1_*NB*(14,2)+(1−*p*_1_)*NB*(0.5,2) and *p*_2_*NB*(5,1)+(1−*p*_2_)*NB*(1,1), respectively. We have chosen these distributions so as to have different IP efficiencies (namely, *IPE*_1_ = 0.9996 and *IPE*_2_ = 0.9732). The plot gives the average BIC value, over 100 iterations, for the model which does not make the assumption of equal probabilities (grey line) versus the model which does make this assumption (black line). The x-axis shows the true *p*_2_−*p*_1_ value for the different simulations, where we fix *p*_1_ = 0.05 and vary *p*_2_ between 0.05 and 0.06. Despite the different IP efficiencies, it is clear how the BIC measure manages to distinguish between the case when *p*_1_ = *p*_2_ and the case when this assumption is not satisfied. The simulation shows further how there is a small margin of error for values of *p*_2_ very close to, but not exactly equal to, *p*_1_.

We have checked whether the assumption of an equal number of binding sites is appropriate for CBP and p300 from the experiments considered in this paper. Of the three comparisons reported in Table 4, the BIC measure suggests that p300 and CBP can be assumed to have an equal number of binding sites at time 0 (both from the Ramos and Wang experiments), but this assumption is not appropriate at time 30 (BIC${}_{p_{1}\neq p_{2}}$ - BIC${}_{p_{1}=p_{2}}=-8034.47$.) Table 5 compares the results of the analyses at time 0. The *p*_1_ ≠ *p*_2_ column shows the results from Table 4, where different probabilities of enrichment are assumed for the different proteins at the different time points. The *p*_1_ = *p*_2_ column gives the results from the new analysis, where *p*_1_ and *p*_2_ are constrained to be equal in the estimation procedure. The results show how the two approaches lead to different results. In particular, fewer regions are detected for the more efficient experiments at the same false discovery rate. Our interpretation of this is that, in the absence of replicates, when one compares two experiments on two different proteins with two different antibodies being used (and consequently different efficiencies of the experiments), it is difficult to estimate accurately the parameter *p*_*c*_ as well as accounting for IP efficiency. Indeed, the estimated *p*_*c*_ values from the more flexible approach, with *p*_1_≠*p*_2_, tend to be quite different in these cases, against expectation (e.g. 0.0305 for CBPT0 and 0.0414 for p300T0 from Table 1). Particularly in these situations, including in the model the assumption of a similar number of binding profiles returns a better estimation of the probability of a region being enriched and consequently it is expected to return a more robust detection of the truly differential binding regions.

**Table 5:** **Number of differentially bound regions at 5% FDR when making an assumption of the same number of binding sites for the two proteins (*p***_***1***_*** = p***_***2***_**), compared to the case when this assumption is not made (*p***_***1***_*** ≠ p***_***2***_**); the last column reports the difference in the BIC values of the two models (a positive difference means a better fit for the model that assumes *p***_***1***_*** = p***_***2***_**)**

**Conditions**	**# Regions bound only by CBP**		**# Regions bound only by p300**		**BIC difference**
	*p*_1_ ≠ *p*_2_	*p*_1_ = *p*_2_	*p*_1_ ≠ *p*_2_	*p*_1_ = *p*_2_	BIC${}_{p_{1}\neq p_{2}}$ - BIC${}_{p_{1}=p_{2}}$
T0	9	11	2726	1277	16172.34
Wang	3069	2630	142	142	3267.04

## Conclusions

Different antibodies are used for ChIP-seq experiments for different proteins, and these have different levels of specificity. On top of this, different ChIP efficiencies are observed even for replicated experiments on the same protein. This results in different signal to background ratios for ChIP-seq generated data, and consequently, in a different percentage of expected enriched and non-enriched regions. We have used simple arguments to show how this is the case, how the ChIP efficiency of an experiment can be quantified from the data and how different ChIP efficiencies for different experiments can lead to misleading biological conclusions if not accounted for in the statistical analysis. This is shown both for the detection of enriched regions and of differentially binding regions, for which a new test is proposed. In the exposition, we focus on the detection of broad regions, such as those marked with histone modifications, and we do not use any information about peak-shape or reads from opposite strands.

We have used a mixture of negative binomial distributions to present the results in this paper. One important point of the paper is that a mixture model approach, such as the one presented here, allows to account for the ChIP-efficiency of an experiment: less efficient experiments are modelled by more overlapping signal/background mixtures than more efficient experiments. In our results, we fitted this model to count data on 1000 bp-size windows. The relatively large window size is motivated by the fact that the mixture model considered here does not account for Markov properties in the data. More sophisticated statistical models of ChIP-seq data, such as HMMs [24] or random fields models [26], or more sophisticated distributions, such as zero-inflated Poisson or negative binomials distributions, e.g. [23,25], can be used within the same framework described in this paper, and are currently under investigation. Similarly more robust estimates of background distributions can be used, e.g. [3,23]. Current research is looking at an extension of the joint model approach presented in this paper to one where read-mappability and GC-content are directly included in the model specification. Furthermore, most of the available normalization methods, e.g. [5-7], work with a pre-defined set of enriched regions and often make use of control experimental data to further improve the identification of enriched regions. The regions detected by the method proposed in this paper could be further used as part of existing normalization procedures.

A second important point of the paper is that estimation of the parameters of the mixture model is performed jointly, from all the available data. In particular, the knowledge of experiments being technical or biological replicates puts some constraints in the parameter space: the parameter *p*_*c*_ that is discussed in the paper is the same for all technical and biological replicates, as these share naturally the same binding profile. This parameter, as well as all the other parameters in the model, are estimated from data by an expected maximum likelihood approach. Given the parameter estimates, the final point of the paper is to show how these can be appropriately used to make a decision about which regions in the genome are enriched, and which are differentially bound in the case of two proteins.

We use real ChIP-seq data on two histone modifiers, p300 and CBP, to show how a joint modelling approach for ChIP-seq data, which properly accounts for the different ChIP efficiencies, is able to identify a larger number of enriched regions than a standard approach, where individual models are fitted to individual experiments and the results of individual analyses are subsequently combined. The regions identified by the joint modelling approach have been validated by TSS overlap and ChromHMM and have generally shown similar enrichment of chromatin features to the regions detected by individual analyses. Additional a priori biological knowledge, such as the expectation of a same number of binding for two different proteins, can also be included in the model and is found to return more realistic numbers of differentially bound regions, with a smaller number of regions bound by the protein where a more efficient experiment is conducted and therefore an expectation of a smaller number of false positives. Further work will be conducted to validate these regions biologically.

The methods described in this paper are implemented in the R package enRich, which is available in CRAN. The input to the main function in this package is count data for a number of bins and a number of experiments, together with information about which experiments are replicates, which experiments are thought to have the same number of binding profiles, which two proteins (if available) should be compared for differential enrichment, and an FDR cut-off for the selection of regions. The output of the function is a list of enriched regions for each protein and each condition and the list of differentially bound regions at the specified FDR cut-off.

## Methods

### The data: pre-processing and validation

The ChIP-seq data on p300 and CBP analysed in this paper was generated from two different labs [13,14]. In [14], CBP and p300 binding is profiled in human T98G cells at time point 0 (T0), where cells are serum starved and where CBP or p300 is restricted to a limit set of genes, and at 30 minutes after stimulation with tetradecanoyl phorbol acetate (T30). For the latter condition, there are two technical replicates (T301 and T302) and it is known that the ChIP efficiency in the second replicate is higher than in the first. In [13], CBP and p300 binding was evaluated in resting CD4+ T cells. We will use the protein names followed by T0, T301 and T302 to refer to the six experiments of [14], use T30 for the combination of T30-1 and T30-2 results and use Wang followed by the protein names to refer to the two experiments in [13].

All sequence reads were aligned to the human genome (build hg18) using BWA version 0.5.9 with default settings. We divide the whole genome into 1000 base pair windows and summarise the raw counts for each window by the number of tags whose first position is in the window. To account for a possible mappability problem [10], we delete the bins which are not covered by any of the experiments mentioned above, resulting in 7.67*%* bins deleted in total. Furthermore, we exclude from the analysis genomic regions that have been found to exhibit anomalous or unstructured read counts (http://hgdownload.cse.ucsc.edu/goldenPath/hg18/encodeDCC/wgEncodeMapability/wgEncodeDukeRegionsExcluded.bed6.gz) [33]. The 2,832,221 remaining regions are considered for the analysis. All the results for enriched and differentially bound regions are given in terms of these 1000bp bins and are provided as Additional file 1. These bins could be further processed by joining consecutive bins into regions.

Overlap with Transcription Start Sites (TSSs) was assessed in Galaxy (https://main.g2.bx.psu.edu), using the first (plus strand) and the last (minus strand) positions of UCSC annotated genes. We consider a bin as containing a TSS when there is at least 1bp overlap with an annotated TSS. Enrichment of the detected regions with chromatin features was assessed using ChromHMM [31]. The method is based on a hidden Markov model, which takes as input the binary vector of enriched and not-enriched regions, obtained from the method described in this paper at a specified FDR cutoff, and gives as output the predicted state for each region. We consider a model with 4 states, as we find that these are enough to capture the diversity of the detected regions in terms of chromatin features enrichment. The resulting predicted states are evaluated for enrichment using a number of external annotations. In particular, we use the Broad ChromHMM classification, available from the UCSC genome browser, and select the following categories: RefSeq exons, silent DNA (Heterochromatin), promoters ready to start transcription (PoisedPromoter), active and weak promoters (ActivePromoter and WeakPromoter, respectively), strong and weak enhancers (StrongEnhancer and WeakEnahncer, respectively).

### The joint latent mixture model: parameter estimation

We take the following steps to estimate the parameters of interest of the mixture model. In order to simplify the notation, we describe the general process without using subscripts *c*,*j*,*i*. We will describe the case of replicates more in detail in the next section. 

1. We choose a grid of values for the offset *k* from 0 to some user defined largest minimum observable tag count, for which we set a default of 10. The parameters of the mixture distributions depend on the choice of *k*.

2. Since *X*_*m*_ is unobserved, we use an EM algorithm to estimate the parameters Θ = (*p*,*θ*^*S*^,*θ*^*B*^) for a fixed value *k*. The complete log likelihood for counts *Y* and unobserved indicators *X* is given by

$$\begin{array}{lll} \;l(Y,X|\Theta ) & =\log(P(Y,X|\Theta )\; & \;\\ & =\log(P(Y|X,\Theta )+\log(P(X|\Theta ))\; & \;\\ & =\overset{M}{\underset{m=1}{\sum }}\{I(X_{m}=1)[\log p+\log P(Y_{m}|X_{m}=1,\theta^{S})]\; & \;\\ & \;+I(X_{m}=0)[\log(1-p)+\log P(Y_{m}|X_{m}=0,\theta^{B})]\}. \end{array}$$

Then the E- and M-steps for the *t*th iteration are as follows:

E-step: Expectation of Likelihood

$$\begin{array}{ll} Q(\Theta |\Theta^{\left(t\right)})=E_{X|Y,\Theta^{\left(t\right)}}l(Y,X|\Theta ) & \; \end{array}$$

where

$$\begin{array}{lll} \;\tau_{1,m}^{\left(t\right)} & =E(X_{m}=1|Y_{m}=y_{m},\Theta^{\left(t\right)})\; & \;\\ & =P(X_{m}=1|Y_{m}=y_{m},\Theta^{\left(t\right)})\; & \;\\ & =\frac{p^{\left(t\right)}f(y_{m}-k|\theta^{S(t)})}{p^{\left(t\right)}f(y_{m}-k|\theta^{B(t)})+(1-p^{\left(t\right)})f(y_{m}|\theta^{B(t)})}\; & \;\\ \;\tau_{0,m}^{\left(t\right)} & =P(X_{m}=0|Y_{m}=y_{m},\Theta^{\left(t\right)})=1-\tau_{1,m}^{\left(t\right)}. & \; \end{array}$$

From this,

$$\begin{array}{lll} \;Q(\Theta |\Theta^{\left(t\right)}) & =\overset{M}{\underset{m=1}{\sum }}\tau_{1,m}^{\left(t\right)}[\log p+\log f(y_{m}|\theta^{S})]\; & \;\\ & \;+\tau_{0,m}^{\left(t\right)}[\log(1-p)+\log f(y_{m}|\theta^{B})].\; & \; \end{array}$$

*M-step: Maximisation*:

$$\begin{array}{ll} \Theta^{\left(t+1\right)}=\underset{\Theta }{\mathrm{argmax}}\;Q(\Theta |\Theta^{\left(t\right)}). & \; \end{array}$$

3. We calculate the marginal likelihood functions for each pair of offset *k* and mixture parameters Θ and choose the pair which gives the largest likelihood values.

### The special cases of poisson and negative binomial

When analysing deep-sequencing data, it is quite common to consider either a Poisson or a Negative Binomial (NB) distribution for the mixture components. In what follows, we give more details on the EM-algorithm implementation in the case of mixtures of Poisson and NB distributions, respectively.

In the *t*th iteration, we maximise the expected likelihood and set the parameters for the next iteration. For the *p* parameter:

$$\begin{array}{lll} \;p^{\left(t+1\right)} & =\underset{p}{\mathrm{argmax}}\{\overset{M}{\underset{m=1}{\Sigma }}\;\;\tau_{1,m}^{\left(t\right)}\log(p)+\overset{M}{\underset{m=1}{\Sigma }}\;(1\;-\;\overset{\left(t\right)}{\underset{1,m}{\tau }})\log(1-p)\}\; & \;\\ & =\frac{1}{M}\overset{M}{\underset{m=1}{\Sigma }}\tau_{1,m}^{\left(t\right)}.\; & \; \end{array}$$

For the other parameters, we need to distinguish the case of Poisson and NB distributions. If signal and background follow Poisson distributions with parameters *λ*_*S*_ and *λ*_*B*_, respectively, we have

$$\begin{array}{lll} \;\lambda_{S}^{\left(t+1\right)} & \;\;=\;\;\underset{\lambda_{S}}{\mathrm{argmax}}\{\overset{M}{\underset{m=1}{\Sigma }}\tau_{1,m}^{\left(t\right)}[(y_{m}-k)\log\lambda_{S}-\lambda_{S}]\}\; & \;\\ & \;\;=\overset{M}{\underset{m=1}{\Sigma }}\tau_{1,m}^{\left(t\right)}y_{m}/\overset{M}{\underset{m=1}{\Sigma }}\tau_{1,m}^{\left(t\right)}-k\; & \;\\ \;\lambda_{B}^{\left(t+1\right)} & \;\;=\;\;\overset{M}{\underset{m=1}{\Sigma }}\tau_{0,m}^{\left(t\right)}y_{m}/\overset{M}{\underset{m=1}{\Sigma }}\tau_{0,m}^{\left(t\right)}.\; & \; \end{array}$$

If both signal and background follow *NB*(*μ*,*ϕ*) distributions, where

$$\text{NB}(\mu ,\phi )=\frac{\Gamma (y+\phi )}{\Gamma (y+1)\Gamma (\phi )}{\left(\frac{1}{1+\mu /\phi }\right)}^{\phi }{\left(\frac{\mu }{\mu +\phi }\right)}^{y},$$

then, similarly to before, we have

$$\begin{array}{llll} \;\mu_{S}^{\left(t+1\right)} & = & \underset{\mu_{S}}{\mathrm{argmax}}\{\overset{M}{\underset{m=1}{\Sigma }}\tau_{1,m}^{\left(t\right)}[-\phi_{S}\log(1+\mu_{S}/\phi_{S})\;\\ & + & (y_{m}-k)\log\left(\frac{\mu_{S}}{\mu_{S}+\phi_{S}}\right)]\}=\frac{\overset{M}{\underset{m=1}{\Sigma }}\overset{\left(t\right)}{\underset{1,m}{\tau }}y_{m}}{\overset{M}{\underset{m=1}{\Sigma }}\overset{\left(t\right)}{\underset{1,m}{\tau }}}-k\;\\ \;\mu_{B}^{\left(t+1\right)} & = & \frac{\overset{M}{\underset{m=1}{\Sigma }}\tau_{0,m}^{\left(t\right)}y_{m}}{\overset{M}{\underset{m=1}{\Sigma }}\tau_{0,m}^{\left(t\right)}}.\; & \;\\ & \; \end{array}$$

And for the overdispersion parameters, $\phi_{S}^{\left(t+1\right)}$ is set as the *ϕ* value that maximises:

$$\begin{array}{lll} \;\overset{M}{\underset{m=1}{\sum }}\tau_{1,m}^{\left(t\right)} & \left[\log(\Gamma (y_{m}\;-\;k\;+\;\phi_{S}))\;-\;\log(\Gamma (y_{m}\;-\;k\;+\;1))\;-\;\log(\Gamma (\phi_{S}))\right)\; & \;\\ & \left(-\phi_{S}\log\;\left(1+\frac{\mu_{S}}{\phi_{S}}\right)+(y_{m}-k)\log\left(\frac{\mu_{S}}{\mu_{S}+\phi_{S}}\right)\right],\; & \; \end{array}$$

and $\phi_{B}^{\left(t+1\right)}$ is set as the *ϕ* value that maximises

$$\begin{array}{lll} & \overset{M}{\underset{m=1}{\sum }}\tau_{0,m}^{\left(t\right)}\left[\log(\Gamma (y_{m}\;+\;\phi_{B}))\;-\;\log(\Gamma (y_{m}\;+\;1\;))\right)\; & \;\\ & \left(\;-\log(\Gamma (\phi_{B}))\;-\;\phi_{B}\log\;\left(1\;+\frac{\mu_{B}}{\phi_{B}}\right)\right)\; & \;\\ & \left(\;+y_{m}\log\;\left(\frac{\mu_{B}}{\mu_{B}\;+\;\phi_{B}}\right)\;\right].\; & \; \end{array}$$

Given that no closed-form solutions can be found for the *ϕ* parameters, we use the optim function in R for this optimization.

### Combining information from replicates in the detection of enriched regions

In this section, we show how the framework described above can be used for the joint analysis of technical and biological replicates. Since replicates are made at the same condition *c*, the latent binding profiles *X*_*mc*_ are the same for these experiments, and consequently also the parameter *p*_*c*_.

Including this assumption in the model is expected to lead to a more robust detection of the enriched regions, particularly when different IP efficiencies are observed for each experiment. This framework would be suited also to the case when different antibodies are used for the different replicates, such as experiments on the same protein conducted in different laboratories.

In what follows, we give the details of the EM algorithm in the presence of replicates. More specifically, in the E-step, the joint log-likelihood function of replicates $Y_{c11},\cdots \;,Y_{\text{cJ}n_{J}}$ and latent variable *X*_*c*_ is given by

$$\begin{array}{lllll} & \;l({\text{Y}}_{c11},\cdots \;,{\text{Y}}_{\text{cJ}n_{J}},{\text{X}}_{c}|\Theta )\; & \;\; & \; & \;\\ & \;=\;\;\overset{M}{\underset{m=1}{\sum }}\{I(X_{\text{mc}}=1)\underset{j,i}{\sum }[\log p_{c}+\log P(Y_{\text{mcji}}|X_{\text{mc}}=1,\theta^{S})]\; & \; & \;\\ & \;+I(X_{\text{mc}}\;=\;0)\underset{j,i}{\sum }[\log(1\;-\;p_{c})\;+\;\log P(Y_{\text{mcji}}|X_{\text{mc}}\;=\;0,\theta^{B})]\}.\; & \; & \; \end{array}$$

Given a fixed offset *k*_*cji*_ for each experiment, replicates share a common *τ*^(*t*)^ term, which is defined as

$$\begin{array}{lll} \tau_{1,m}^{\left(t\right)} & = & E(X_{\text{mc}}=1|Y_{\text{mcji}},\Theta^{\left(t\right)},k_{\text{cji}})\\ & = & \frac{p_{c}^{\left(t\right)}\underset{j,i}{\prod }P(y_{\text{mcji}}\;-\;k_{\text{cji}}|\theta_{\text{cji}}^{S(t)})}{p_{c}^{\left(t\right)}\underset{j,i}{\prod }P(y_{\text{mcji}}\;-\;k_{\text{cji}}|\theta_{\text{cji}}^{S(t)})\;+\;(1\;-\;p_{c}^{\left(t\right)})\underset{j,i}{\prod }P(y_{\text{mcji}}|\theta_{\text{cji}}^{B(t)})}\\ \tau_{0,m}^{\left(t\right)} & = & 1-\tau_{1,m}^{\left(t\right)}. \end{array}$$

Using the estimates of the mixture model parameters from the bin counts *Y*, we can predict each bin as being enriched or not under condition *c* by computing the posterior probability of the latent variable, that is

$$\begin{array}{lll} P(X_{\text{mc}} & = & 1|y_{\text{mcji}},{\hat{\Theta }}_{\text{cji}},{\hat{k}}_{\text{cji}},{\hat{p}}_{c})\\ & = & \frac{{\hat{p}}_{c}\underset{j,i}{\prod }P(y_{\text{mcji}}-{\hat{k}}_{\text{cji}}|{\hat{\theta }}_{\text{cji}}^{S})}{{\hat{p}}_{c}\underset{j,i}{\prod }P(y_{\text{mcji}}-{\hat{k}}_{\text{cji}}|{\hat{\theta }}_{\text{cji}}^{S})+(1-{\hat{p}}_{c})\underset{j,i}{\prod }P(y_{\text{mcji}}|{\hat{\theta }}_{\text{cji}}^{B})}\\ P(X_{\text{mc}} & = & 0|y_{\text{mcji}},{\hat{\Theta }}_{\text{cji}},{\hat{k}}_{\text{cji}},{\hat{p}}_{c})\;=\;1\;\;-\;P(X_{\text{mc}}\;=\;1|y_{\text{mcji}},\;{\hat{\Theta }}_{\text{cji}},{\hat{k}}_{\text{cji}},{\hat{p}}_{c}). \end{array}$$

Note that a single probability of enrichment is derived under condition *c* by combining all replicates under this condition.

As a final step in the analysis, we set a threshold on the posterior probabilities to decide whether a bin is enriched or not under a particular condition. Different criteria can be used to set this cut-off. In BayesPeak [24], an 0.5 cut-off is used, whereby each region is assigned to the state with the highest posterior probability. In this paper, as in [34], we use a cut-off corresponding to a specific value of the expected posterior false discovery rate. If *D* is the number of enriched regions corresponding to a particular cut-off on the posterior probabilities, then the expected false discovery rate for this cut-off is given by

(2) $$\begin{array}{ll} \bar{\text{FDR}}=\frac{\underset{m\;\text{enriched}}{\Sigma }\;P(X_{\text{mc}}=0|y,{\hat{\Theta }}_{\text{cji}},{\hat{k}}_{\text{cji}},{\hat{p}}_{c})}{D}. & \; \end{array}$$

This allows to account for the different IP efficiencies in the detection of enriched regions.

### Detection of differentially bound regions

We formally develop a test of differential binding based on the probability

$$\begin{array}{lll} \;P(X_{m1}\neq X_{m2}) & \;\;=\;\;P(X_{m1}=0|Y_{1})P(X_{m2}=1|Y_{2})\; & \;\\ & \;+P(X_{m1}=1|Y_{1})P(X_{m2}=0|Y_{2})\; & \; \end{array}$$

where *P*(*X*_*mc*_ = 0|*Y*_*c*_) is the probability that the *m*th bin is enriched for protein *c*, estimated by the model described above from all the data on protein *c*, at the same time point.

W define *Z* as a random variable indicating the common binding profiles of two proteins, that is *Z*_*m*_ = 1 if *X*_*m*1_ ≠ *X*_*m*2_ and *Z*_*m*_ = 0 if *X*_*m*1_ = *X*_*m*2_. Then, *P*(*Z*_*m*_ = 0) = *P*(*X*_*m*1_ = *X*_*m*2_) and a cutoff can be set on the probabilities of differential binding by controlling a predefined FDR value, using the same formula defined in (2).

### Estimating ChIP efficiencies

We derive a formal method to quantify IP efficiencies of a ChIP-seq experiment based on the mixture model that best fits the data. Let *Y*^*S*^ and *Y*^*B*^ be the random variables representing the counts in a signal and background region, respectively. We estimate IP efficiency by calculating the probability that the counts in the background region are lower than those in the signal regions. Formally,

(3) $$\begin{array}{ll} P(Y^{B}<Y^{S})=\int_{0}^{\infty }\int_{0}^{y}f_{B}(z)f_{S}(y)\text{dzdy}, & \; \end{array}$$

with *f*_*B*_ and *f*_*S*_ the background and signal densities, respectively, and assuming independence in the counts at different locations.

This quantity varies between 0.5 and 1, namely 0.5 for perfectly overlapping components (inefficient experiment) and 1 for perfectly separated components (efficient experiment). Real estimates will vary between these two extremes, the higher this value, the more efficient the experiment is. The formula can be used to estimate ChIP efficiency for mixture models with any two distributions and could be easily extended to more than two mixture components.

## Competing interests

The authors declare that they have no competing interests.

## Authors’ contributions

PtH and VV initiated the study. YB, VV and EW developed the statistical methodology. YB implemented the algorithm. PtH assisted in the development of the methodology and the interpretation of the results. PtH performed the biological validation. YB and VV wrote the manuscript. All authors read and approved the final manuscript.

## Supplementary Material

Additional file 1 **Enriched and differentially bound regions.** Excel file listing the enriched regions and the differentially bound regions identified by the joint and individual analyses (corresponding to Tables 1, 3, 4 and 5 of the main manuscript). Click here for file

## References

[B1] Robertson G Hirst M Bainbridge M Bilenky M Zhao Y Zeng T Euskirchen G Bernier B Varhol R Delaney A Thiessen N Griffith O He A Marra M Snyder M Jones S Genome-wide profiles of STAT1 DNA association using chromatin immunoprecipitation and massively parallel sequencing Nat Methods 2007 4 8 651 657 10.1038/nmeth1068 17558387

[B2] Kidder B Hu G Zhao K ChIP-Seq: technical considerations for obtaining high-quality data Nat Immunol 2011 12 10 918 922 10.1038/ni.2117 21934668 PMC3541830

[B3] Diaz A Park K Lim D Song J Normalization, bias correction, and peak calling for ChIP-seq Stat Appl Genet Mol Biol 2012 11 3 Article 9 22499706 10.1515/1544-6115.1750 PMC3342857

[B4] Mendoza-Parra MA Sankar M Walia M Gronemeyer H POLYPHEMUS: R package for comparative analysis of RNA polymerase II ChIP-seq profiles by non-linear normalization Nucleic Acids Res 2011 40 4 e30 22156059 10.1093/nar/gkr1205 PMC3287170

[B5] Liang K Keleş S Detecting differential binding of transcription factors with ChIP-seq Bioinformatics 2012 28 121 122 10.1093/bioinformatics/btr605 22057161 PMC3244766

[B6] Nair N Sahu A Bucher P Moret B ChIPnorm: a statistical method for normalizing and identifying differential regions in histone modification ChIP-seq libraries PLoS ONE 2012 7 8 e39573 10.1371/journal.pone.0039573 22870189 PMC3411705

[B7] Shao Z Zhang Y Yuan G Orkin S Waxman D MAnorm: a robust model for quantitative comparision of ChIP-Seq data sets Genome Biol 2012 13 3 R16 10.1186/gb-2012-13-3-r16 22424423 PMC3439967

[B8] Song Q Smith A Identifying dispersed epigenomic domains from ChIP-seq data Bioinformatics 2011 27 6 870 871 10.1093/bioinformatics/btr030 21325299 PMC3051331

[B9] Taslim C Huang K Huang T Lin S Analyzing ChIP-seq Data: Preprocessing, Normalization, Differential Identification, and Binding Pattern Characterization Next Generation Microarray Bioinformatics Methods Mol Biol 2012 802 275 291 10.1007/978-1-61779-400-1_18 22130887

[B10] Rozowsky J Euskirchen G Auerbach R Zhang Z Gibson T Bjornson R Carriero N Snyder M Gerstein M PeakSeq enables systematic scoring of ChIP-seq experiments relative to controls Nat Biotechnol 2009 27 66 75 10.1038/nbt.1518 19122651 PMC2924752

[B11] Lan X Bonneville R Apostolos J Wu W Jin V W-ChIPeaks: a comprehensive web application tool for processing ChIP-chip and ChIP-seq data Bioinformatics 2011 27 3 428 430 10.1093/bioinformatics/btq669 21138948 PMC3031039

[B12] Kalkhoven E CBP and p300: HATs for different occasions Biochem Pharmacol 2004 68 6 1145 55 10.1016/j.bcp.2004.03.045 15313412

[B13] Wang Z Zang C Cui K Schones D Barski A Peng W Zhao K Genome-wide mapping of HATs and HDACs reveals distinct functions in active and inactive genes Cell 2009 138 1019 1031 10.1016/j.cell.2009.06.049 19698979 PMC2750862

[B14] Ramos Y Hestand M Verlaan M Krabbendam E Ariyurek Y van Dam H van Ommen G den Dunnen J Zantema A’t Hoen P Genome-wide assessment of differential roles for p300 and CBP in transcription regulation Nucleic Acids Res 2010 38 16 5396 5408 10.1093/nar/gkq184 20435671 PMC2938195

[B15] Wilbanks E Facciotti M Evaluation of algorithm performance in ChIP-seq peak detection PLoS ONE 2011 5 7 e11471 20628599 10.1371/journal.pone.0011471 PMC2900203

[B16] Micsinai M Parisi F Strino F Asp P Dynlacht B Kluger Y Picking ChIP-Seq peak detectors for analyzing chromatin modification experiments Nucleic Acids Res 2012 40 9 e70 10.1093/nar/gks048 22307239 PMC3351193

[B17] Fejes A Robertson G Bilenky M Varhol R Bainbridge M Jones S FindPeaks 3.1: a tool for identifying areas of enrichment from massively parallel short-read sequencing technology Bioinformatics 2008 24 15 1729 1730 10.1093/bioinformatics/btn305 18599518 PMC2638869

[B18] Nix D Courdy S Boucher K Empirical methods for controlling false positives and estimating confidence in ChIP-Seq peaks BMC Bioinformatics 2008 9 523 10.1186/1471-2105-9-523 19061503 PMC2628906

[B19] Ji H Jiang H Ma W Johnson D Myers R Wong W An integrated software system for analyzing ChIP-chip and ChIP-seq data Nat Biotechnol 2008 26 11 1293 1300 10.1038/nbt.1505 18978777 PMC2596672

[B20] Jothi R Cuddapah S Barski A Cui K Zhao K Genome-wide identification of in vivo protein-DNA binding sites from ChIP-seq data Nucleic Acids Res 2008 36 16 5221 5231 10.1093/nar/gkn488 18684996 PMC2532738

[B21] Zhang Y Liu T Meyer C Eeckhoute J Johnson D Bernstein B Nussbaum C Myers R Brown M Li W Model-based analysis of ChIP-Seq (MACS) Genome Biol 2008 201 R137 18798982 10.1186/gb-2008-9-9-r137 PMC2592715

[B22] Humbrug P Helliwell C Bulger D Stone G ChIPseqR: analysis of ChIP-seq experiments BMC Bioinformatics 2011 1471–2105 12 39 10.1186/1471-2105-12-39 PMC3045301 21281468

[B23] Kuan P Chung D Pan G Thomson J Stewart R Keles S A statistical framework for the analysis of ChIP-Seq data J Am Stat Assoc 2011 106 495 891 903 10.1198/jasa.2011.ap09706 PMC4608541 26478641

[B24] Spyrou C Stark R Lynch A Tavare S BayesPeak: Bayesian analysis of ChIP-seq data BMC Bioinformatics 2009 10 299 10.1186/1471-2105-10-299 19772557 PMC2760534

[B25] Qin Z Yu J Shen J Maher C Hu M Kalyana-Sundaram S Yu J Chinnaiyan A HPeak: an HMM-based algorithm for defining read-enriched regions in ChIP-seq data BMC Bioinformatics 2010 11 369 10.1186/1471-2105-11-369 PMC2912305 20598134

[B26] Mo Q A fully Bayesian hidden Ising model for ChIP-seq data analysis Biostatistics 2012 13 113 128 10.1093/biostatistics/kxr029 21914728

[B27] Koerber R Rhee H Jiang C Pugh B Interaction of transcriptional regulators with specific nucleosomes across the Saccharomyces genome Mol Cell 2009 35 6 889 902 10.1016/j.molcel.2009.09.011 19782036 PMC2760215

[B28] Fan X Lamarre-Vincent N Wang Q Struhl K Extensive chromatin fragmentation improves enrichment of protein binding sites in chromatin immunoprecipitation experiments Nucleic Acids Res 2008 36 19 e125 e125 10.1093/nar/gkn535 18765474 PMC2577354

[B29] Blahnik K Dou L O’Geen H McPhillips T Xu X Cao A Iyengar S Nicolet C Ludascher B Korf I Farnham P Sole-Search: an integrated analysis program for peak detection and functional annotation using ChIP-seq data Nucleic Acids Res 2010 38 3 e13 10.1093/nar/gkp1012 19906703 PMC2817454

[B30] Bardet A He Q Zeitlinger J Stark A A computational pipeline for comparative ChIP-seq analyses Nature Protoc 2012 7 1 45 61 22179591 10.1038/nprot.2011.420

[B31] Ernst J Manolis K Discovery and characterization of chromatin states for systematic annotation of the human genome Nat Biotechnol 2010 28 8 817 827 10.1038/nbt.1662 20657582 PMC2919626

[B32] Xu H Wei C Lin F Sung W An HMM approach to genome-wide identification of differential histone modification sites from ChIP-seq data Bioinformatics 2008 24 20 10.1093/bioinformatics/btn402 18667444

[B33] Hoffman M Ernst J Wilder KASP Harris R Libbrecht M Giardine B Ellenbogen P Bilmes J Birney E Hardison R Dunham I Kellis M Noble W Integrative annotation of chromatin elements from ENCODE data Nucleic Acids Res 2012 41 2 827 841 23221638 10.1093/nar/gks1284 PMC3553955

[B34] Broët P Richardson S Detection of gene copy number changes in CGH microarrays using a spatially correlated mixture model Bioinformatics 2006 22 8 911 918 10.1093/bioinformatics/btl035 16455750

